# *Trypanosoma cruzi* Infection at the Maternal-Fetal Interface: Implications of Parasite Load in the Congenital Transmission and Challenges in the Diagnosis of Infected Newborns

**DOI:** 10.3389/fmicb.2019.01250

**Published:** 2019-06-07

**Authors:** Patricia L. Bustos, Natalia Milduberger, Bibiana J. Volta, Alina E. Perrone, Susana A. Laucella, Jacqueline Bua

**Affiliations:** ^1^ Instituto Nacional de Parasitología “Dr. Mario Fatala Chaben” – ANLIS C. G. Malbrán, Buenos Aires, Argentina; ^2^ Centro de Altos Estudios en Ciencias Humanas y de la Salud (CAECIHS), Universidad Abierta Interamericana, Buenos Aires, Argentina

**Keywords:** *Trypanosoma cruzi*, mother-to-child transmission, parasitemia, infected pregnant women, congenitally infected infants, early diagnosis

## Abstract

*Trypanosoma cruzi* is the protozoan unicellular parasite that causes Chagas disease. It can be transmitted from infected mothers to their babies *via* the connatal route, thus being able to perpetuate even in the absence of Triatomine insect vectors. Chagas disease was originally endemic in Central and South America, but migration of infected women of childbearing age has spread the *T. cruzi* congenital infection to non-endemic areas like North America, Europe, Japan, and Australia. Currently, 7 million people are affected by this infection worldwide. This review focuses on the relevance of the *T. cruzi* parasite levels in different aspects of the congenital *T. cruzi* infection such as the mother-to-child transmission rate, the maternal and fetal immune response, and its impact on the diagnosis of infected newborns. Improvements in detection of this parasite, with tools that can be easily adapted to be used in remote rural areas, will make the early diagnosis of infected children possible, allowing a prompt trypanocidal treatment and avoiding the current loss of opportunities for the diagnosis of 100% of *T. cruzi* congenitally infected infants.

## Epidemiology of the Connatal Chagas Disease

The American trypanosomiasis, or Chagas disease, is caused by the protozoan parasite *Trypanosoma cruzi*, which affects about 6–7 million people worldwide, with most of the cases in Latin America (WHO | Chagas disease (American trypanosomiasis), 2019).

Given the great success in the control of *Triatoma infestans*, after which Brazil, Paraguay, Uruguay, and Chile were free of *T. cruzi* vectorial transmission, and appropriate control of blood supply that interrupted parasite infection through blood transfusion in most endemic countries, interruption of mother-to-child *T. cruzi* transmission became the new challenge in research and in public health policies. Around 9,000 babies are born to *T. cruzi*-infected mothers each year, and it is estimated that 1.1 million women of childbearing age are infected with *T. cruzi* in 21 countries from Mexico to Argentina, where this neglected tropical disease is endemic (WHO | Chagas disease (American trypanosomiasis), 2019).

Currently, *T. cruzi* infection is globally distributed and has been increasingly detected in countries where vector transmission is absent, mainly due to migration of infected individuals from Latin America. Among the non-endemic countries, the United States is home to the largest number of Chagas infection cases, estimated to be more than 300,000, of which a small number of cases were reported as autochthonous vector-borne transmission in the southern US (Bern et al., 2011; Manne-Goehler et al., 2016), whereas the number of *T. cruzi*-infected people has exceeded 100,000 in Europe (Strasen et al., 2013). It has had a smaller impact in Canada, Australia, and Japan (Buekens et al., 2008; Imai et al., 2014; Jackson et al., 2014). There is a substantial proportion of *T. cruzi*-infected women of childbearing age and congenitally infected infants among the Latin American migrants (Soriano-Arandes et al., 2016). Conversely, *T. cruzi* transmission through blood transfusion or organ transplants are of less epidemiological importance, since non-endemic countries with large immigrant populations have begun to intervene in blood-borne *T. cruzi* transmission (Gascon et al., 2010).

The outcome of congenital infection with *T. cruzi* is due to the result of complex interactions among the parasite, the placenta and the immune responses of the mother and the fetus, and studies about the mechanism of congenital infection are scarce. Understanding these relationships would help in successfully preventing congenital transmission of the parasite or facilitate better access to diagnosis and treatment of the newborns, which would eventually contribute to decreasing the number of cases of this disease around the world.

## *T. Cruzi* Mother-To-Child Transmission

It has been reported that *T. cruzi* maternal-fetal transmission occurs in about 1–12% of the pregnancies, taking into account reports with the largest number of infected pregnant women studied in endemic areas (Russomando et al., 1998; Torrico et al., 2005; De Rissio et al., 2010; Salas Clavijo et al., 2012; Bua et al., 2013). The rate of parasite transmission is variable in different countries: 6% in Argentina, 4.1% in Bolivia, and 4.3% in Paraguay (Carlier and Truyens, 2015), 1.7% in Brazil (Martins-Melo et al., 2014), and between 0.8 and 4.08 in Mexico (Cardoso et al., 2012; Montes-Rincón et al., 2016), with an average rate of around 5% (Howard et al., 2014). The wide variation in the reported rates is probably due to studies performed in different areas with and without vector transmission, with heterogeneous populations, experimental conditions and different diagnostic methods.

## Maternal Parasitemia and Vector Exposure

The vertical transmission rate of *T. cruzi* is different in areas with or without the presence of insect vectors, as geographic regions where the disease is endemic are twice as likely to have congenital transmission, compared to the countries free of transmission vectors, 5.0 vs. 2.7% respectively, according to studies performed mainly in Spain (Howard et al., 2014). It was intuitive that parasite load would be enhanced under continuous vector exposure in endemic areas, increasing the risk of parasite congenital transmission (Dias et al., 2002; Torrico et al., 2006). However, it was also demonstrated that infected women living in houses under active vector control had significantly higher parasite loads compared to those women who lived in infested houses (Sánchez Negrette et al., 2005; Rendell et al., 2015). This is probably due to repeated parasite inoculations, which induce an enhanced immune response that helps to control the parasite levels (Rendell et al., 2015).

## Maternal Parasitemia and Risk of Connatal Parasite Transmission

A correlation between high parasitemia in pregnant women and the risk of maternal-fetal *T. cruzi* transmission was observed when the blood from mothers of infected children showed a higher frequency of positive parasite hemocultures (Hermann et al., 2004). A higher parasitemia was also observed in the blood buffy coats of women that transmitted the parasite to their offspring compared to those who did not (Salas et al., 2007; Brutus et al., 2010). A higher frequency of vertical parasite transmission was observed in *T. cruzi* acute infection, which is usually associated with an increased parasitemia (Moretti et al., 2005). Among the studies that quantified parasite load in seropositive pregnant women, mothers of infected babies had significantly higher parasitemia, compared to the mothers of non-infected babies (Virreira et al., 2007; Bern et al., 2009; Bua et al., 2012; Kaplinski et al., 2015; Rendell et al., 2015). When the parasitic load, quantified by quantitative polymerase chain reaction (qPCR), was correlated with the parasite transmission rate in 128 *T. cruzi*-infected pregnant women from Bolivia, researchers found that 31.3% of women with a high parasite load (35 Pe/mL or more) delivered infected children, compared to 15.4% in women with a moderate parasite load (between 1 and 34 Pe/mL), and 0% in women with a parasite load of less than 1 Pe/mL (Rendell et al., 2015). Similar results were obtained in another study in Spain, with migrants from Bolivia and Paraguay, where 31% of pregnant women with detectable *T. cruzi* DNA by conventional PCR delivered infected children, whereas a 0% parasite transmission rate was observed in babies born to chronic infected mothers with negative PCR findings (Murcia et al., 2013).

A high *T. cruzi* parasite load was also detected in patients co-infected with HIV (Rosemberg et al., 1992), and a 100% parasite transmission rate was observed in children born to mothers with reactivated Chagas disease, due to immunosuppression in four different studies (Freilij and Altcheh, 1995; Nisida et al., 1999; Scapellato et al., 2009; Agosti et al., 2012).

Congenital *T. cruzi* infection cannot be prevented during pregnancy as there are no studies on the possible teratogenic effects in pregnant women treated with trypanocidal drugs, benznidazole or nifurtimox. However, six different retrospective studies showed that no congenital infection was detected in infants delivered from a total of 243 infected women that had been treated with benznidazole or nifurtimox prior to pregnancy, in childhood or even in early adulthood (Sosa-estani et al., 2009; Murcia et al., 2013, 2017; Fabbro et al., 2014; Moscatelli et al., 2015; Álvarez et al., 2017).

Altogether, research in this field has strongly supported that parasitemia during pregnancy is a key factor for *T. cruzi* connatal transmission, considering that 100% of the infants were born infected with *T. cruzi* when their mothers displayed high parasitemia during pregnancy. On the other hand, 100% of the children born to drug-treated women or women with naturally very low or no parasitemia were uninfected (Murcia et al., 2013; Rendell et al., 2015). These studies reinforce the notion that decreasing the parasite load might be beneficial in avoiding congenital infection, and supports the idea that either a specific trypanocidal treatment or a possible preconceptional therapeutic vaccine with *T. cruzi* recombinant proteins in near future (Dumonteil et al., 2019) should be offered to women of childbearing age who could potentially transmit the infection to their babies.

## Parasitemia and Maternal Immune Response

Interferon-gamma (IFN-γ) and tumor necrosis factor (ΤΝF) are key mediators that control *T. cruzi* infection. IFN-γ activates monocytes/macrophages and stimulates, in synergy with TNF-α, the generation of nitric oxide which kills the parasite (Carlier and Truyens, 2015).

Regarding the maternal immune response during pregnancy, it has been observed that mothers that gave birth to infected children have decreased plasma levels of TNF-α (Cardoni et al., 2004; García et al., 2008) and moderately decreased circulating levels of soluble TNF receptor 1 (sTNF-R1), compared to mothers of uninfected children. Soluble TNF receptors downregulate the biological activity of TNF-α by competing with its membrane receptors (García et al., 2008).

A decrease in production of IFN-γ, in response to parasite antigens, was found in the blood cells derived from the mothers of infected children before and after delivery. However, similar levels of intracellular IFN-γ, within CD3^+^ cells derived from both groups of infected mothers, were found after polyclonal activation, indicating that they have a comparable ability to produce IFN-γ. Mothers that gave birth to infected children also showed decreased percentages of activated T lymphocytes and monocytes, compared to those who did not transmit the infection to their offspring (Hermann et al., 2004).

Another study that compared cytokine production in *T. cruzi*-infected women that gave birth to uninfected children showed that the mothers with detectable parasitemia presented increased levels of IFN-γ and TNF-α in peripheral, placental and cord blood (Cuna et al., 2009), compared to infected mothers with undetectable parasitemia. These results indicate that, when a higher parasite load is associated with a more robust but pro-inflammatory response, there is no congenital transmission.

Altogether, these findings indicate that the ability to control the infection through an appropriate innate and adaptive immune response against *T. cruzi* to maintain a low parasite load in mothers is associated with lower rates of vertical transmission. Alterations in the control of the inflammatory response may have direct consequences on the congenital transmission and on the children’s immune response. Taking into account the challenges related to congenital Chagas diagnosis, the identification of immunological mediators could be very useful for the development of new biomarkers of vertical transmission risk.

## Parasitemia and Fetal Immune Response

*T. cruzi* infection in pregnant women can induce the activation of T lymphocytes in the fetus *in utero*, as supported by the production of proinflammatory cytokines like interleukin (IL) 1β, IL-6 and TNF-α, in response to *T. cruzi* antigens in uninfected infants born to *T. cruzi*-infected mothers (Vekemans et al., 2000; Hermann et al., 2002; García et al., 2008).

The study of serum cytokines showed a distinct immune profile in congenitally infected infants, with a vigorous innate immune response skewed towards a Th17 profile. Decreased levels of IFN-γ, but increased levels of IL-17A, monokine induced by gamma interferon (MIG) and monocyte chemoattractant protein-1 (MCP-1), were revealed as early predictors of *T. cruzi* infection in the presence of either high or low parasitemia, while *T. cruzi*-infected infants also displayed increased levels of IL-6 and IL-17F, but only in the presence of low parasitemia (Volta et al., 2016).

These demonstrations of a distinct and polarized profile of cytokines and chemokines in the circulation of infants born to *T. cruzi*-infected mothers, and its correlation with the newborn parasite load, reinforce the role of the immune system in restricting the severity of this parasitic infection, preventing the morbidity and mortality of a possible congenital Chagas disease.

## Parasite Diversity and the Placental Barrier

*T. cruzi* parasites display genetic differences that have been defined by molecular markers and can be differentiated into six discrete typing units or DTUs (TcI to TcVI), with a localized geographical distribution (Zingales et al., 2012). Although efforts have been made to correlate the different *T. cruzi* DTUs with parasite virulence or clinical manifestations in humans, there has not been any clear association so far (Del Puerto et al., 2010).

*T. cruzi* parasites from almost all DTUs, except TcIV, have been found in babies born to infected mothers. TcV is the predominant DTU reported in the congenital cases in the Southern cone countries of Latin America (Burgos et al., 2007; Virreira et al., 2007; Corrales et al., 2009). In our laboratory, 38 parasite isolates were obtained from 382 *T. cruzi-*infected pregnant women, which represents 10% positive hemocultures in this group. All of the isolated parasites were identified as TcV, among which only six belonged to mothers who have gave birth to infected children (Bua et al., 2012); thus, we were not able to associate any parasite DTU with connatal transmission. Nevertheless, it would be interesting to study the genetic differences between parasites isolated from mothers who did not transmit the infection in two or even three different pregnancies and those parasites isolated from infected children (Bua et al., 2013), looking at other biomarkers that would probably help in discriminating the divergences in virulence and pathogenicity under the DTU classification.

Many studies have tried to mimic the human maternal-fetal *T. cruzi* transmission in experimental models with rare offspring infections (Carlier and Truyens, 2015), but some studies with TcI, TcII and TcVI strains revealed that infected pups were obtained only from acutely infected mice with Y and Tulahuen strains which were TcII and TcVI, respectively (Cencig et al., 2013). This suggests that the connatal transmission in experimental models were related more to mice parasitemia than DTU differences. It was possible to obtain a congenital transmission rate of 3.7% in chronically infected mice with the *T. cruzi* strain RA (TcVI), although maternal parasitemia in those mice was significantly higher than the mice infected with K98 clone/TcI, from which no congenitally infected offspring were obtained (Solana et al., 2002).

Another interesting study on the genetic response of the placenta in chronic experimental infections in mice compared the virulence of *T. cruzi* K98 clone with an isolated parasite from a congenitally infected child (VD/TcVI), and demonstrated that the murine placental infection with the VD isolated parasite was associated with upregulation of genes related to components of the innate immune system and IFN-γ. Even so, no congenital transmission was observed in pups born to infected mice with VD nor K98 parasites (Juiz et al., 2017). The VD/TcVI parasite proved to be more infective in the human trophoblast-derived cell line BeWo compared to the *T. cruzi* Y strain/TcII (Medina et al., 2018), probably due to a higher virulence and placental tropism, as it was isolated from a human case of congenital infection (Risso et al., 2004). Nevertheless, no significantly different infection levels could be observed on placental explants with a *T. cruzi* isolated from a congenitally-infected newborn (Lucky, TcII/VI) compared to the Tulahuen strain (TcVI), although the isolated parasite Lucky showed a greater survival rate in a deleterious placental milieu (Triquell et al., 2009). It was demonstrated that a high inoculum of these two parasites resulted in increased infection of placental explants, producing structural and physiological changes through nitric oxide synthase and oxidative-nitrosative stress of the placental barrier (Triquell et al., 2018).

The human placenta forms an anatomical barrier between the maternal blood and fetal tissue, and when infected by *T. cruzi,* a reorganization of the extracellular matrix occurs (Duaso et al., 2012), and a differential expression of pro-inflammatory and immune-modulating cytokines has been observed in infected human placental explants (Castillo et al., 2018), confirming the important role of this organ in avoiding parasite infectivity (Liempi et al., 2014; Díaz-Luján et al., 2016; Juiz et al., 2017).

## Parasitemia and Diagnosis in Congenitally Infected Infants

Since most *T. cruzi*-infected pregnant women and children are asymptomatic, this parasite infection can go undetected. Additionally, there is a current under-diagnosis of this infection due to losses of opportunities in the prenatal care and proper child follow-up by the health system surveillance programs (Carlier et al., 2015).

The diagnosis of *T. cruzi* congenitally infected children under 8–10 months of age relies primarily on the detection of the parasite, usually live parasites in blood by microscopic methods, as specific antibodies are usually transferred by their seropositive mothers. Only when parasitological assays fail to detect the infection are infants required to be monitored over time for the detection of parasite-specific antibodies, which confirm additional cases of *T. cruzi* infection when maternal antibodies disappear (De Rissio et al., 2010).

In most of the Latin American countries, an early diagnosis of infants born to *T. cruzi*-infected pregnant women relies on the direct examination of the buffy coat from fresh blood samples collected in microhematocrit heparinized tubes or microtubes. This micromethod has limited analytical sensitivity (40–50 parasites/mL) and strongly depends on trained operators (Freilij and Altcheh, 1995; De Rissio et al., 2010), due to the fact that this method needs a minimal 30 min of microscopic observation per sample. Since the micromethod only detects 40–60% of congenitally infected newborns, it is necessary to perform additional serological tests at 8–10 months of age, a period in which around 40–60% of the children do not complete the follow-up for the final diagnosis of this infection (De Rissio et al., 2010; Bua et al., 2013).

## Molecular Approaches For the Parasitological Diagnosis of the Connatal *T. Cruzi* Infection

*T. cruzi* nucleic acid amplification by PCR has been utilized since 1998 (Russomando et al., 1998) for the detection of *T. cruzi* in congenitally infected babies, offering a higher sensitivity and specificity than parasitological methods involving direct microscopic examination of blood buffy coat samples (Schijman et al., 2003; Virreira et al., 2003; Mora et al., 2005). Later, qPCR technology was developed (Piron et al., 2007; Virreira et al., 2007; Duffy et al., 2009, 2013; Ramírez et al., 2015) and was able to detect 0.85 or 0.43 parasite equivalents per mL (Pe/mL) of satellite DNA and kinetoplastid DNA, respectively, providing more sensitivity than the conventional PCR technique (Cura et al., 2017). qPCR emerged as a potential tool for an accurate and early diagnosis of congenital *T. cruzi* infection (Virreira et al., 2007; Bua et al., 2013). However, a positive amplification of parasitic DNA in newborns could be ambiguously interpreted as a result of maternal parasite DNA debris not related to the passage of live parasites (Virreira et al., 2007), and thus for a positive DNA amplification in babies close to birth it would not be confirmative of parasite infection (Carlier et al., 2015). This represents a disadvantage over microscopic detection methods, which rely on the observation of viable and motile parasites (Freilij et al., 1983). To avoid misinterpretations, PCR diagnosis would be more reliable at 1 month after delivery (Bua et al., 2013) or for the confirmation of diagnosis with a subsequent blood sample (Murcia et al., 2017).

It is important to highlight that qPCR requires highly equipped laboratories and robust quality controls, frequently found in urban areas or reference health centers but rarely available in maternities or primary point of care units in endemic areas (Porrás et al., 2015; Messenger and Bern, 2018; Picado et al., 2018). In fact, PCR is not included as a tool for diagnosis of congenital *T. cruzi* infection in the Latin American guidelines, with the exception of Chile, although its use is sometimes recommended (Picado et al., 2018). In Argentina, this molecular technique is in the process of being transferred to different laboratories of the national public health network (Cura et al., 2017).

Other molecular methods that could be implemented for the *T. cruzi* diagnosis are the techniques based on isothermal amplification of DNA. These methods overcome the needs for specialized PCR equipment and have been proven to amplify the *T. cruzi* DNA successfully. Loop-mediated isothermal amplification (LAMP) can be performed at a constant temperature of 60–65°C with a simple heat-block (Besuschio et al., 2017; Rivero et al., 2017), and the recombinase polymerase amplification (RPA) can be run at 37–42°C (Castellanos-Gonzalez et al., 2018) with a sensitivity similar to that of the quantitative PCR amplification (Besuschio et al., 2017; Jimenez-Coello et al., 2018). These new molecular approaches await the necessary standardization and validation, but, as with all molecular techniques, the main issue is that parasite DNA amplification cannot be performed without purification of DNA from patient blood samples, which cannot be performed easily in health centers in rural areas, as it requires experienced operators, infrastructure and the necessary quality controls recommended by good practice guidelines.

## Parasitemia in *T. Cruzi* Infected-Children at a 1-Year Follow-Up Study

Parasitemia levels in infants congenitally infected with *T. cruzi* are significantly higher at birth than in their infected mothers, who are usually in the chronic phase of this infection (Schijman et al., 2003; Virreira et al., 2007; Bern et al., 2009; Bua et al., 2012), and no correlation has been observed between the parasitemia of pregnant women and their babies (Bua et al., 2012).

Parasite load was quantified by qPCR in 51 infected babies born to *T. cruzi*-infected mothers in a retrospective study. These babies were grouped according to the time and method in which congenital infection was diagnosed during 1-year follow-up after delivery. A group of 19 newborns diagnosed by micromethod at 1 month showed the highest median parasitemia, around 1,700 Pe/mL. The infected infants that came back for a second parasitological diagnosis at 6 months of age showed a median parasitemia of around 20 Pe/mL in the sample obtained at 1 month of age, which was under the threshold of the micromethod sensitivity. This group of 10 infants could be diagnosed by microscopy at 6 months of age because parasitemia increased up to 500 Pe/mL. In the infants (22/51) negative for the first and second parasitological control, who required serological diagnosis at around 1 year of age, the median parasite load was 5,800 and 20 Pe/mL in the blood samples obtained at 1, 6, and 12 months after delivery, respectively. This study helped to understand the differences among diverse groups of *T. cruzi* congenitally infected children during 1 year follow-up in centers where molecular techniques are not available (Bua et al., 2013). An infected child that is not diagnosed at 8–10 months after delivery and not treated will experience a drastic decrease of parasitemia, indicating the transition from the acute phase to the chronic phase of the *T. cruzi* infection, and most importantly, will be excluded from the possibility of being treated with trypanocidal drugs.

Although DNA amplification has shown great sensitivity for the detection of cases of *T. cruzi* mother-to-child transmission, qPCR does not detect 100% of congenital cases, and in case of negative PCR findings, it is necessary to detect the congenital infection by serology at 8–10 months of age. Quantitative PCR was able to detect *T. cruzi* infection in 50/51 babies in the first control visit, and we did not observe any false positive PCR in the babies diagnosed by micromethod at 1 month after delivery (Bua et al., 2013). The only *T. cruzi-*infected infant that could not be diagnosed by qPCR at 20 days nor at 6 months of age was infected by a TcI *T. cruzi* parasite that was isolated by hemoculture at 7 months of age, a period in which the specific anti-*T. cruzi* serology was also positive (Volta et al., 2018).

## Search of Biomarkers For the Early Serological Diagnosis of the Connatal *T. Cruzi* Infection

As mentioned, the detection of anti-*T. cruzi* specific antibodies in infants born to seropositive mothers can be performed when they are 8–10 months of age, when maternal passively transferred antibodies are no longer detectable (Moya et al., 1989). A positive serological result at this time is a conclusive diagnosis for the *T. cruzi* congenital infection in infants where previous parasitological methods failed to detect the parasite. Unfortunately, only 40–60% of congenitally infected children complete the required 1-year follow-up (Sosa-Estani, 2005; De Rissio et al., 2010). Efforts are being made to find specific serological markers to diagnose this infection at an earlier stage and overcome the loss of opportunities to detect 100% of the *T. cruzi* infected children as soon as they are born.

The *T. cruzi* Shed Acute Phase Antigen (SAPA) (Affranchino et al., 1989) proved to be a reliable and highly sensitive marker for the early parasite detection of congenital *T. cruzi* infection (Reyes et al., 1990). An ELISA test available in Paraguay detects anti-SAPA IgG antibodies in children born to infected mothers at 3 months of age (Russomando et al., 2010). The anti-SAPA IgG levels in binomial blood samples from seropositive mothers and their babies also allowed for diagnosis in 90.5% of the *T. cruzi-*infected children at around 1 month of age (Volta et al., 2015) by subtracting the anti-SAPA OD value of the mother from the one in the child (Mallimaci et al., 2010). We observed a positive correlation between parasitemia levels in mothers and infants, evaluated by qPCR, and the anti-SAPA IgG antibody titers detected by ELISA, which more likely accounts for the secretion of SAPA antigen by the trypomastigotes in the bloodstream (Volta et al., 2015).

Trypomastigote secreted/excreted (TESA) protein bands of 120–200 kDa were blotted on membranes (Umezawa et al., 1996) and recognized by anti-SAPA IgM antibodies in acute and congenital *T. cruzi*-infected children, but with lower sensitivity than qPCR (Messenger et al., 2017). Western blotting with TESA antigen helped discriminate the chronic maternal infection by detecting IgG bound to TESA (a single 150–160 kDa band) from the blood of newborns with acute infection, highlighting the presence of four to six SAPA-specific protein bands between 120 and 200 kDa on the IgM TESA-blot (Noazin et al., 2018). Although the sensitivity of the anti-SAPA IgM on TESA blots reached 80% in *T. cruzi-* infected newborns (Noazin et al., 2018), the immunodetection of membrane strips has several issues of reproducibility and standardization in care units outside urban areas (Messenger and Bern, 2018).

The SAPA antigen has been considered as a promising biomarker for the diagnosis of *T. cruzi* infection, and some new approaches for the development of diagnostic assays which include detection of SAPA along with other antigenic determinants in single multiplex assays, to confirm the *T. cruzi* infection in humans, are being developed (Granjon et al., 2016). SAPA has also been included in the design of chimeric molecules, named as CP1 and CP3, which were sensitive enough to circumvent inconclusive diagnosis in subjects with serodiscordant findings (Peverengo et al., 2018). These multi-epitope constructs are currently being tested for an improved detection of congenital infected newborns (Dr. Ivan Marcipar, personal communication).

Another recent development was to span *T. cruzi* linear B-cell epitopes and design antigenic short peptides to achieve an accurate diagnosis of this infection in chronic human samples by ELISA (Mucci et al., 2017). The next step is to extend this approach with the aim of an early accurate diagnosis for congenital infection (Dr. Fernán Agüero, personal communication).

In summary, the current search for highly sensitive serological diagnostic tests based on multiple antigenic determinants in multiplex assays could offer the possibility to detect *T. cruzi*-infected children born to seropositive women. A prompt diagnosis may prevent dropout during the 1-year serological follow-up after delivery required for the accurate diagnosis of *T. cruzi* infection. Ideally, serological diagnosis for early parasite detection in newborns could be available as a lateral flow immunochromatographic test, with an affordable cost for public health systems, easily performed by operators with minimal training, without the need of any specialized and costly equipment, no reagent preparations, with immediate results and easy adaptability for use in primary health care facilities, public hospitals or maternities in endemic area. As established by WHO, *T. cruzi* infection is curable if treatment is initiated soon after infection (Carlier et al., 2011; WHO | Chagas disease (American trypanosomiasis), 2019). Many reports have shown that benznidazole and nifurtimox treatments are well tolerated in children and resulted in undetectable parasite load (Russomando et al., 1998; Blanco et al., 2000; Schijman et al., 2003; Altcheh et al., 2005; Luquetti et al., 2005).

Infants who fail to complete the required follow-up period for parasite diagnosis will be deprived of access to immediate drug treatment and parasite clearance and will become *T. cruzi-*infected adults. It is crucial to develop improved, rapid and simple diagnostic methods for a timely detection of *T. cruzi* congenital infection, soon after birth and before the newborn leaves the care unit, especially in rural areas where access to the health system can be limited.

## Conclusion

Mother-to-child transmission of *T. cruzi* infection represents a challenge in controlling parasite dissemination in endemic and non-endemic regions. Parasitemia in infected women plays a key role in congenital Chagas outcome, as it directly affects transmission rate and maternal and fetal protective immune response against the parasite. In fact, decreasing parasite load by trypanocidal treatment administered to women of childbearing age proved to be highly efficient in avoiding congenital infection. Parasite levels in congenitally infected newborns have a direct impact on their diagnosis, so it is crucial to develop improved diagnostic methods to facilitate access to treatment.

## Author Contributions

PB, NM, BV, AP, SL, and JB wrote the manuscript and approved its final version.

## Conflict of Interest Statement

The authors declare that the research was conducted in the absence of any commercial or financial relationships that could be construed as a potential conflict of interest.
